# Patient-controlled encrypted genomic data: an approach to advance clinical genomics

**DOI:** 10.1186/1755-8794-5-31

**Published:** 2012-07-20

**Authors:** Yannis J Trakadis

**Affiliations:** 1 Department of Medical Genetics, Montreal Children’s Hospital-McGill University Health Centre, 2300 Tupper, Montreal, QC, Canada

**Keywords:** Genomic, Genome, Exome, Encrypted, Sequencing, Clinic, Integration, I-MPOS, I-MPOSE

## Abstract

**Background:**

The revolution in DNA sequencing technologies over the past decade has made it feasible to sequence an individual’s whole genome at a relatively low cost. The potential value of the information generated by genomic technologies for medicine and society is enormous. However, in order for exome sequencing, and eventually whole genome sequencing, to be implemented clinically, a number of major challenges need to be overcome. For instance, obtaining meaningful informed-consent, managing incidental findings and the great volume of data generated (including multiple findings with uncertain clinical significance), re-interpreting the genomic data and providing additional counselling to patients as genetic knowledge evolves are issues that need to be addressed. It appears that medical genetics is shifting from the present “phenotype-first” medical model to a “data-first” model which leads to multiple complexities.

**Discussion:**

This manuscript discusses the different challenges associated with integrating genomic technologies into clinical practice and describes a “phenotype-first” approach, namely, “Individualized Mutation-weighed Phenotype Search”, and its benefits. The proposed approach allows for a more efficient prioritization of the genes to be tested in a clinical lab based on both the patient’s phenotype and his/her entire genomic data. It simplifies “informed-consent” for clinical use of genomic technologies and helps to protect the patient’s autonomy and privacy. Overall, this approach could potentially render widespread use of genomic technologies, in the immediate future, practical, ethical and clinically useful.

**Summary:**

The “Individualized Mutation-weighed Phenotype Search” approach allows for an incremental integration of genomic technologies into clinical practice. It ensures that we do not over-medicalize genomic data but, rather, continue our current medical model which is based on serving the patient’s concerns. Service should not be solely driven by technology but rather by the medical needs and the extent to which a technology can be safely and effectively utilized.

## Background

The revolution in DNA sequencing technologies over the past decade has made it feasible to sequence an individual’s whole genome at a relatively low cost [1], [2]. The potential value of the information generated by genomic technologies for medicine and society is enormous. However, personal genomic testing may not be ready for widespread clinical use [3]. Analysis, interpretation and management of the data generated from exome sequencing (ES), which targets slightly more than 1% of the entire genome, have already proven to be a challenge in the research context for mendelian disorders [4]. One can only imagine the level of complexity that will result from trying to incorporate whole genome sequencing (WGS) into *clinical* practice, especially in dealing with common diseases characterized by complex inheritance. Nonetheless, the low cost of WGS may encourage its early adoption creating “a million dollar headache” [5].

First, the extent to which it is possible to obtain meaningful patient informed-consent to large-scale genomic analysis has been called into question [6], [7]. For instance, genomic approaches increase the chance of discovering incidental findings or results with uncertain clinical significance. Mutations with incomplete penetrance, mutations in novel genes with unknown function, and genetic variants responsible for very small increases in disease risk are typical challenging examples. This information is difficult to conceptualize and can generate emotional distress over disease risk even among healthy individuals [8]. Hence, during counselling, the potential benefits for individuals need to be weighed against the potential harm and the individuals’ right not to know.

Currently, genetic risk assessment is restricted to individuals at increased risk based on family history or clinical presentation, which may be indirectly ensuring the necessary genomic background for the pathogenic role of a variant. Recent reports suggest that on average, each person is heterozygous for approximately 50–100 variants classified by the Human Gene Mutation Database (HGMD) as causing inherited disorders and of approximately 250 to 300 loss of function variants in annotated genes [9]. With what degree of certainty can we predict the impact of even known pathogenic variants when they are identified as incidental findings [10], [11]? Moreover, data from personal genome sequencing is a powerful personal identifier challenging the traditional mechanisms of protection. This raises concerns about privacy, confidentiality and potential genetic discrimination [12].

Responsibly managing the very large amounts of genetic information produced is another key challenge to overcome [13]. As stated above, the significance of the data generated from ES/WGS varies from clearly clinically relevant (e.g. “pathogenic mutations”) to data of unknown clinical significance (“variants of unknown significance”, VUS). The evaluation and management of novel variants identified using ES/WGS require substantial time and cost. Expertise is needed for analysis of the complex genomic data but there are not enough clinical geneticists to interpret results from wide-spread whole-genome sequencing and to provide follow-up information and clinical care [14,15]. Moreover, return of genomic results requires substantial financial resources for genetic counselling of patients, particularly in the context of the dynamic, continuously evolving, nature of the interpretation of ES/WGS results [16]. The practical challenge of the duty to re-contact patients as knowledge changes over time is already experienced in medical genetics secondary to the clinical implementation of array-comparative genomic hybridization (array CGH) and research studies using ES. This will be exponentially more complex in the context of large-scale *clinical* applications of ES, and especially WGS [17].

Overall, premature integration of ES/WGS into clinical care may lead to a cascade effect [18] and strain the health-care resources in a disproportionate, unjustified fashion [19]. The immediate, actionable, applications of the information derived from genomic technologies are limited at present time [20]. Moreover, approximately 20% of all human genes and, thus, many of the genes with immediate clinical application have patents on them [21]. Reporting mutations identified by genome sequencing in such genes has not been resolved (e.g. the Prometheus and Myriad Genetics cases) [22], [23].

It appears that medical genetics is shifting from the present “phenotype-first” medical model to a “data-first” model which leads to multiple complexities (see Table 1). To accelerate the integration of ES/WGS into clinical practice a substantial shift in thinking is required. This manuscript outlines a novel approach which can help address the above-mentioned challenges. The described “phenotype-first” medical model proposes that ES/WGS could incrementally be implemented in day-to-day clinical practice in the immediate future, as long as our focus remains on diseases which, at the time of the evaluation, have been genetically well characterized.

**Table 1:** Challenges of integrating ES/WGS in clinical practice

**Meaningful patient informed-consent may not be feasible**	
*Complex medical and social implications of the test results,*	
*Possibility of incidental findings,*	
*Multiple findings of uncertain clinical significance,*	
*Insurance companies do not reimburse pre-testing counselling for ES/WGS*	
*Multiple issues to discuss leading to prohibitive requirements in time & resources*	
**Potential emotional distress over disease risk even among healthy individuals**	
**Genomic information is a powerful personal identifier**	
*Raising concerns about privacy, confidentiality, genetic discrimination*	
**Very large amounts of genetic information generated**	
*Limited number of clinical geneticists for data interpretation and clinical care*	
*Substantial time and cost for data analysis and genetic counselling*	
*Dynamic/evolving nature of the interpretation as new knowledge is gained*	
*Duty to re-contact patients as knowledge changes over time*	
**Shift from the present “phenotype-first” medical model to a “data-first” model.**	
*Currently, genetic risk assessment is restricted to individuals at increased risk based on family history or clinical presentation (ensuring the necessary genomic background), or newborn screening programs meeting specific criteria.*	
*Can we predict the impact of even known pathogenic variants outside this setting?*	
*Is there adequate evidence to suggest that a departure from these standards will be beneficial to society?*	

## Discussion

### Overview of current clinical genetic practice

In current clinical practice, in order to identify the genetic variant responsible for a patient’s disease, first there is recognition of a more or less specific phenotype followed by molecular testing of a series of relevant genetic loci, individually or in small sets [24]. There exist a variety of online databases which aim to catalogue all known diseases with a strong genetic component (e.g. mendelian genetic syndromes) and link, at least some of, these diseases to the relevant genes in the human genome. A very well known example of such a database which is freely available is OMIM [Online Mendelian Inheritance in Man; http://omim.org/. Typically, to aid in the diagnosis of rare genetic syndromes, a physician would perform a database search using patient-specific information derived from the medical evaluation performed in clinic. The clinician would then verify the search-results and clinically prioritize genetic testing for the disease that he/she considers most likely. Clinical genetic tests are often very expensive, so typically only one gene, or occasionally a group of pre-selected genes typically responsible for one disease, will be analyzed at a given time. If the first test is negative, sequential testing of the genes responsible for the other, less likely but still clinically suspected, genetic diseases usually follows.

Once a genetic change is identified, the physician evaluates the variant for pathogenicity. Some genetic variants can be unequivocally interpreted as pathogenic or normal variants on the basis of extensive clinical experience [25]. In other cases, clinical experience is insufficient and pathogenicity is inferred based on the variants’ predicted effect on protein stability, function, or conservation. Examples of different criteria used in the clinical interpretation of a genetic variant identified by sequencing include: (1) whether the identified variant has been listed as a non-pathogenic variant, (2) whether the variant has been previously reported to cause disease in the literature and/or databases listing such variants, (3) the nature of the mutation: e.g. a silent or a nonsense mutation, (4) the position affected and whether this position is well-conserved across species, and (5) the presence or absence of the mutation in affected or unaffected family members. Computational inferences, although very useful, are not always reliable. Clinical experience shows that even mutations predicted in-silico with high-confidence to be deleterious may ultimately have no major consequences [26], [27].

At present, genomic sequencing technologies are mainly limited to research. They are typically used to identify the mutant gene which could explain a specific disease not-yet genetically characterized. Criteria similar to the ones mentioned above for the interpretation of clinical test results are used to evaluate the pathogenicity of the multiple genetic variants identified by ES/WGS and their possible role in the phenotype under investigation. ES has proven to be a powerful research tool for gene discovery in rare monogenic syndromes [for instance, [28-31]. As the cost of sequencing drops, the use of WGS is also becoming a research option [32-34]. The genes underlying most unsolved mendelian disorders known will likely soon be elucidated thanks to ES/WGS.

### Individualized Mutation-weighed Phenotype Search

**Figure 1:**
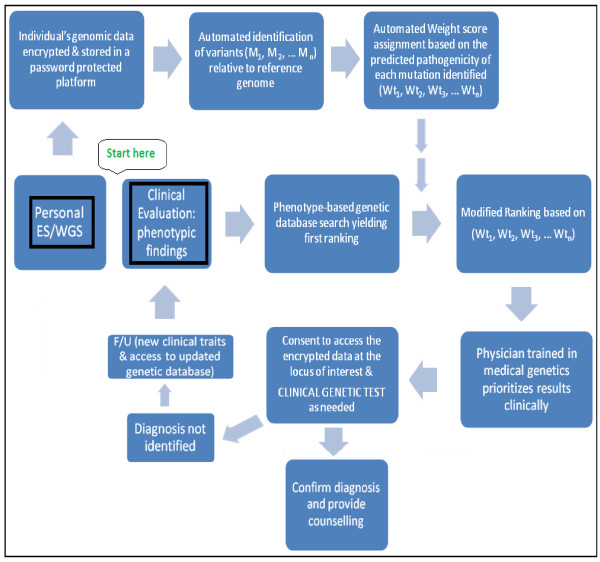
**Schematic overview of I-MPOS, the new clinical genetic approach proposed.** Patients have their exome/genome sequenced and their encrypted data stored on a password-protected platform which remains at the disposal of the individual patient. A patient presents to clinic with a specific medical concern. The physician performs a clinical evaluation and identifies some important features (“phenotype-first” approach). He or she then performs a database search using keywords related to the clinically assessed phenotype, as presently done, thereby providing an initial ranking of possible genetic diseases. This initial ranking is then adjusted by I-MPOSE based on the weight scores automatically assigned to the mutations identified by ES/WGS in the patient’s genes/loci known to be linked to each genetic disease; thus providing a second ranking of the possible genetic diseases. I-MPOSE simultaneously operates on the patient’s encrypted data and on a regularly updated database containing all well characterized genetic diagnoses. It is run during every clinical visit so as to incorporate new findings from clinical evaluation, as well as, new genetic knowledge incorporated in the regularly updated database.

This manuscript proposes a new approach, Individualized Mutation-weighed Phenotype On-line Search (I-MPOS), which can help properly integrate genomic technologies in everyday clinical practice in the immediate future. In the illustrated approach (Figure. 1), patients have their exome/genome sequenced. The sequencing results are stored in an encrypted form on a platform (such as a chip, a memory key, or stored on a cloud) and can be accessed via a password only available to the individual patient. After obtaining authorization, the clinician can temporarily and anonymously upload the patient’s encrypted data to a search engine, namely, Individualized Mutation-weighed Phenotype On-line Search Engine (I-MPOSE). I-MPOSE simultaneously operates on the patient’s encrypted data and on a regularly updated database containing selected genetic diagnoses. Only the diagnoses for which there exists reliable evidence for a pathogenic relationship with at least one confirmed relevant region in the human genome and a well-described, more or less specific, subset of phenotypic characteristics are included in the database.

**Figure 2:**
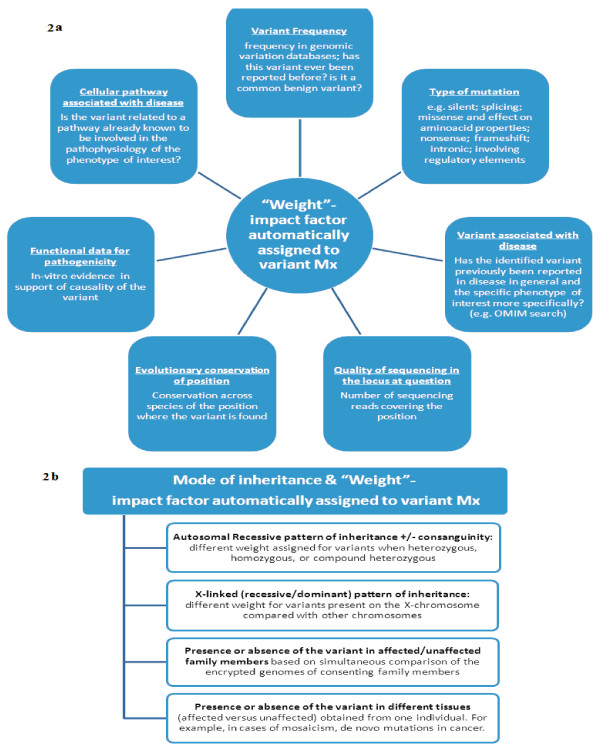
**Examples of factors to be taken into consideration when calculating a variant’s “weight”.** An overall score is automatically calculated by I-MPOSE for each variant identified with ES/WGS by simultaneously taking into consideration different factors. Some examples of such factors are listed above in Figure. 2. Information about the pattern of inheritance can also be factored in the weight assignment process (Figure.2b). The overall score corresponds to the level of certainty for the pathogenicity of each genetic variant identified. An option to allow for adjusting the default parameters is possible through an interactive checklist. The physician can opt to assign a different contribution for a specific parameter (e.g. a much increased contribution for homozygosity when dealing with consanguinity) in the calculation of the variant’s overall score.

**Figure 3:**
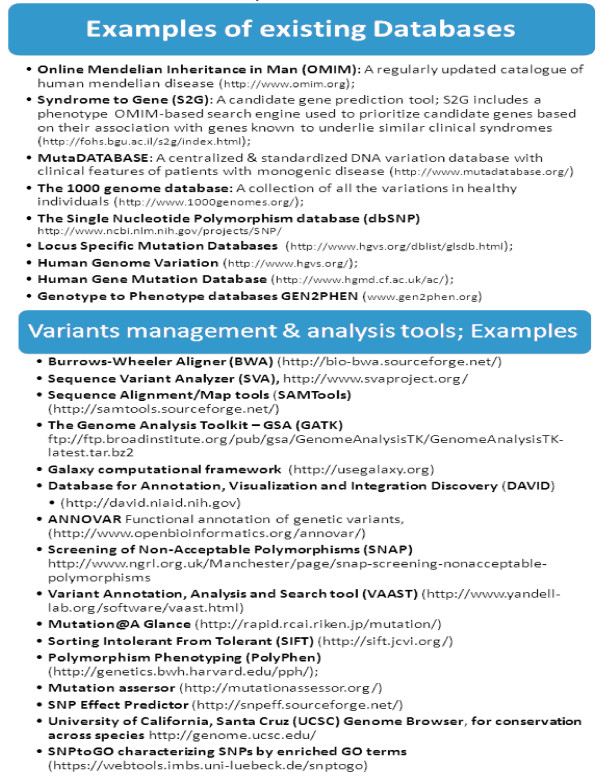
**Examples of ongoing projects & tools illustrating that the necessary infrastructure for I-MPOSE is already available.** Some examples of existing databases for human disease and genetic variation, as well as, tools available for the annotation of variants identified by ES/WGS.

I-MPOSE identifies the genetic changes present in the patient’s sequenced encrypted genome relative to the reference genome and assigns a weight score to each of them based on predetermined criteria integrated in the search algorithm. The pre-set criteria evolve as new knowledge is acquired and are equivalent, at any time, to the criteria used in the clinical evaluation of variants identified after a clinical genetic test (please refer to Figure. 2 for some examples based on current practice). The overall impact score calculated for each genetic variant identified corresponds to the level of certainty for its pathogenicity. The implementation of this approach can take advantage of the numerous databases and tools currently used in the interpretation of genetic variants generated by clinical genetic testing or ES/WGS clinical research (Figure. 3).

For this method to be most useful, the database has to include all known diseases which have been both phenotypically and genetically well characterized, at any given time, and be regularly updated based on research findings and clinical experience. Searching this database while taking into consideration both the patient’s phenotype and his/her entire genomic data when ranking the results could aid enormously in the medical genetic evaluation of patients (please refer to discussion below). In summary, the patient presents to clinic with a specific medical concern. As currently practiced, the physician performs a clinical evaluation and identifies some important features (“phenotype-first” approach). He or she then performs a database search using keywords related to the clinically assessed phenotype, as presently done, thereby providing an initial ranking of possible genetic diseases. This initial ranking of possible genetic diseases is then adjusted by I-MPOSE based on the assigned weight scores for the variants identified by exome/genome sequencing in this patient; thus providing a second ranking of possible genetic diseases based on the said adjustment.

Given that the patient’s personal genome data is encrypted, the physician does not yet know anything about the exact mutations identified by genome/exome sequencing of the patient evaluated. However, after viewing the adjusted ranking results, the physician could provide counselling to the patient and obtain informed consent to have access to the genomic data at the specific locus of interest before sending a sample for clinical genetic testing to confirm the diagnosis.

### EXAMPLE

A patient P referred to genetics for hypotonia suffers from Smith Lemli Opitz (SLO) syndrome. The patient P’s exome has been sequenced. According to the crude data from exome sequencing, patient P has a homozygous mutation, in the *DHCR7* gene, known to cause SLO. The patient is clinically evaluated by a medical geneticist following exome sequencing. The geneticist is not aware of the mutation in the *DHCR7* gene nor that the patient has SLO syndrome. The only positive indications the geneticist identifies as important during the evaluation are “hypotonia” and a “heart defect”. The geneticist performs two searches, a standard search as well as a search with I-MPOSE. The standard search is performed on the previously-mentioned website, OMIM [http://omim.org/].

The OMIM search results for the keywords “hypotonia AND heart defect” garnered 175 hits. SLO based on the search terms had a match score (Y) and was ranked #23 on this list. The I-MPOSE search was also performed on the OMIM website in tandem in conjunction with the patient’s encrypted sequencing data. The geneticist did not look at the ES data per se. He/she simply ran during the search in tandem with the clinical criteria “hypotonia AND heart defect” the ranking system previously described, which took into consideration the automatically assigned impact score of each variant identified by ES. Given that the identified pathogenic mutation in *DHCR7* was assigned by I-MPOSE a high impact score (e.g. X), this time the cumulative score of SLO (Y x X) was relatively higher than that of most other diseases on the list. Hence, SLO was ranked higher on the search results list and the geneticist entertained the diagnosis of SLO. The physician decided to send for *DHCR7* gene clinical testing to confirm the diagnosis. In this example, as per current practice, before undertaking clinical molecular testing the geneticist could have chosen to send a less-expensive non-genetic lab test named a sterol profile, which if positive would have further increased the physician’s a priori suspicion for SLO. Similarly, the physician, after discussing his suspicion of SLO syndrome with the patient (or the family of the patient as appropriate), could have obtained consent to specifically analyze the patient’s encrypted exome sequencing data in the *DHCR7* gene locus. This analysis could be performed in collaboration with the clinical molecular lab director before ultimately pursuing the clinical diagnostic test. A more careful analysis of the changes identified by ES/WGS in this locus would have thus provided further evidence to support that the diagnosis of SLO syndrome needed to be entertained.

### Clinical Implications

Conventional approaches often require multiple genetic tests before a molecular diagnosis is reached and each clinical genetic test is often very expensive. The proposed approach, which follows the existing “phenotype-first” medical model, allows for better prioritization of the genes to be tested in a clinical lab and is overall efficient from a resource, time and personnel point of view both in the clinic and the laboratory.

Based on the lessons learned from the past [35,36], an evidence-based, cautious approach to genomic medicine will be more efficient than premature efforts to integrate these technologies into clinical practice [5]. The I-MPOS approach allows ES/WGS to be used as a screening test rather than a clinical diagnostic test. A confirmatory clinical diagnostic test is still required, thus at least temporarily addressing the issue of patented genes, while ensuring reliable test results. The object of this method is to increase the diagnostic yield of a clinical evaluation in a cost-effective fashion and to decrease the time to diagnosis. For the remaining unresolved cases, the clinician needs to resort back to conventional approaches, which reduces false negatives, as well as, accounts for other known genetic explanations (e.g. epigenetic changes) not accounted for by ES/WGS. The clinician remains responsible for identifying and clinically prioritizing testing for the clinically suspected syndromes using the whole spectrum of clinical genetic testing modalities available. I-MPOSE is, simply, an additional tool at the clinician’s disposal to make the foregoing assessment. Its limitations are minimized as long as qualified medical geneticists perform the evaluation, at least in the early stages of medical genomics, until other health professionals are adequately educated regarding its value, limitations, and appropriate clinical use.

In practice, the clinician does not have access to the patient’s genome/exome sequencing results. The results remain encrypted and password-protected so that the patient retains full control over his/her data and how it is utilised. The patient only needs to consent that the clinician can explore the genetic causes which can potentially explain the specific phenotype/medical-issue at hand. The clinical terms entered in a search are selected based on the diseases of interest for which the family is seeking medical advice. Patients whose diagnosis has not been made will be seen in follow-up, as per present practice, and I-MPOSE will be run during every clinical visit so as to incorporate new findings from clinical evaluation, as well as, new genetic knowledge incorporated in the regularly updated database (Figure.1 for diagram). Hence, I-MPOS addresses many of the challenges associated with integrating genomic technologies into clinical practice (see Table 2).

**Table 2:** Benefits of the I-MPOS approach


-Simplification of pre-test counselling, informed consent and post-test counselling	
-Protection of the patient’s privacy and autonomy	
-Genomic sequencing, even during newborn period, practical, ethical and clinically useful	
-Increased diagnostic yield; cost-effective approach; decreased time to diagnosis	
-Overcomes challenges such as shortage of personnel, the management of the huge amount of data generated, incidental findings, VUS, and duty to re-contact.	
-Incremental integration of genomic technologies into clinical practice.	
-Standardized approach in medical genomics, even where resources are limited	
-Promotion of genomic research	
-Refinement of clinical phenotype: partial matches for known syndromes; biochemical phenotypes	
-Prevents over-medicalization of genomic data while enabling serving the patient’s concerns	
-Progressive education of both the health care personnel and the general public regarding personalized/preventive medicine	

Some individuals are likely to want to learn more from their genomic data as soon as ES/WGS is performed and this could be available in private, if properly regulated. However, in most centers, investing financial recourses for such services would place pressure on an already strained health-care system [37,38]. Until the organisational, ethical, legal, and social issues are properly addressed, and the clinical validity/utility, as well as, the cost-effectiveness of such findings are proven, the clinician’s access to the raw data will be restricted to rare challenging cases in a research setting. Hence, the I-MPOS paradigm helps to standardize care in the field of medical genomics based on realistic expectations which evolve as new knowledge is acquired, thus progressively increasing the quality of care provided to patients. Initially benefits will mostly focus on exome sequencing and rare mendelian conditions frequently encountered by clinical geneticists. The widespread utilization of ES/WGS will subsequently provide us lessons about more complex diseases.

Databases with patient-specific genomic data can store information of patients who decide to have their data stored for future reference or research. These databases will be managed similarly to the guidelines for “biobanks”. With this approach, anonymized exome sequencing data from a great number of consenting individuals will likely be available and help us advance our knowledge of clinical application of genomic technologies. At a clinical level, on top of enabling earlier diagnosis [39], I-MPOS will aid in the refinement of the phenotype of known syndromes. For example, in conventional approaches only patients who fit very closely all or almost all characteristics of a described genetic syndrome are tested for the genes involved. The proposed approach provides for the partial matches of patients to be identified and their characteristics to enrich and refine the spectrum of clinical characteristics of any given syndrome.

### Existing infrastructure and Future Directions

The most central components needed for I-MPOS to be successfully implemented are the availability of well curated databases and the reliability of the “impact score” assignment for each variant identified. To aid in the latter, multiple computational tools exist (some of which are listed in Figure.3). There are also software tools which enable the user to take advantage of a gene filter (e.g. based on quality score, pathway, gene-ontology, OMIM information) and different biological databases when performing the functional annotation. One such example is the Sequence Variant Analyzer (SVA), which is also allowing users to explore the strength of the associations of identified variants with studied traits [40].

As previously stated, computational predictions about the pathogenicity of variants are not always reliable. However, combining the results of different such methods, as well as population and/or family-based data about the cosegregation of the variant with disease, using different statistical methods can be helpful in the calculation of an “overall impact-score” which is more reliable. For instance, Bayesian analysis can be used to factor in results of different computational tools in the overall score. Moreover, relative risk analysis and/or Transmission Disequilibrium Test (TDT) analysis for each variant at a population or family level can also be very useful in predicting a variant’s pathogenicity, particularly when ES/WGS usage becomes wide-spread [41-44]. Freely available statistical software like Family Based Association Test, a.k.a. FBAT, can be useful to this end [45]. In the future, the combination of different bioinformatics and statistical tools will be incrementally more important in the calculation of the “impact score” of each variant. It is also possible that metabolomics will lead to the identification of specific metabolic profiles/signatures associated with specific variants and that the (degree of) biochemical imbalance will constitute an important factor in predicting the pathogenicity, and thus in calculating the variant’s “impact factor”. There are already projects in metabolomics which in the future could be useful to this end [46-48].

In the first steps of the I-MPOS endeavor, though, one needs to be particularly cautious and focus on variants known to be pathogenic based on clinical experience. Already existing guidelines for variant interpretation can be used to classify each variant identified in one of four or six different groups with a different “impact factor” potentially assigned to each group [49,50]. Alternatively, initially the data of already existing databases listing annotated variants and classifying them as pathogenic or not can be the only factors included in the score assignment. Worldwide, ongoing efforts already aim to create comprehensive collections of validated associated variants, as illustrated in Figure.3. Potentially useful curated databases include the Human Gene Mutation Database (HGMD) [51], Diagnostic Mutation Database (DMuDB) [52], MutaDatabase [53], and the ClinVar database [54,55]. The implementation of common methods and clinical standards for data collection and reporting are of great importance for the optimal operation of I-MPOSE. The interpretation of genomic information will change over time as new risks are identified and others are refuted. Hence, the database to be used will need to be updated regularly with all the genetic changes confirmed based on research and clinical experience to cause or predispose to a genetic disease/phenotype. With regards to phenotypic data, an interface with freely available or private databases (such as OMIM, Genereviews, London Medical Databases, or Possum Web) will be needed [56-59]. In order for the search to be optimized, a standardized vocabulary of phenotypic abnormalities encountered in human disease and the semantic relationships between them will be important. The Human Phenotype Ontology (HPO) and Phenomizer could be very useful to this end [60].

In the future, it would be useful if the database used for I-MPOSE included *genes* sorted into “priority tiers” based on the evidence of involvement in each genetic disease (“informatics disease panels” [61]). This could be expanded to include prioritization of genes involved in specific sub-phenotypes (rather than the genetic disease as an entity) like “microcephaly”, “short stature” or even more complex phenotypes such as biochemical profiles. In brief, illnesses, like schizophrenia or Type 1 Diabetes [62-66], with high heritability but complex inheritance, may be shown in the future to constitute a constellation of different pathogenetic processes leading to a similar phenotype. Some of these pathogenetic processes may ultimately have a specific signature not only at the level of the genome, but also at the level of the epigenome, transcriptome or metabolome. If the known such signature profiles are listed in the databases used by I-MPOSE, the data generated at the time of the clinical evaluation from RNA sequencing [67-69] and/or metabolomic profiling [70,71] or even epigenomic changes [72] over time in such a (symptomatic) patient could serve as a phenotypic trait refining the search.

The concept of “informatics disease panels” can also help in the identification of novel genes contributing to known genetic diseases/phenotypes. One can use different methods (see [73-82] for examples) to select candidate genes for each genetic disease/phenotype and also include them in the database, albeit with a lower “priority”, representing the amount of evidence/probability of the gene’s involvement in the respective genetic disease. For any given patient, when a phenotype is ranked highly after I-MPOSE search but no mutations are found in all genes documented to cause the disease, consent to explore the *pre-selected* candidate genes for the phenotype at hand for mutations could be obtained.

In conclusion, the widespread utilization of ES/WGS will subsequently provide us lessons about complex genetic diseases. The weight factors and the assigning process will evolve based on emerging knowledge (e.g. to account for gene-gene or gene-environment interactions in complex diseases [83-89]). As research and genetic knowledge advance, the benefits of this approach will increase exponentially. I-MPOS could ultimately help cautiously drive the paradigm shift to personalized healthcare and eventually preventive medicine.

## Summary

This is an exciting time for medicine: the era of genomic medicine. However, the implementation of ES/WGS in clinical practice should only take place when the challenges posed by ethics and policy issues have been addressed. Unfortunately, it is very likely that the fast pace of development of these technologies and their ability to facilitate diagnosis, to reduce the incidence of deleterious genetic disorders, and to inform therapy will lead to their premature introduction in clinic [90]. As previously discussed, the era of WGS is just around the corner [91]. Already ES is employed by some direct-to-consumer genetic testing companies [19,92]. We now need to prepare the framework required for responsible and successful integration of ES/WGS in patient care; thus ensuring the appropriate and effective usage of genomic information [91]. It is widely recognized that automated systems and clear guidelines will be necessary in this process. Our current approaches need to be reassessed but not changed solely based on the availability of a new technology. For instance, currently genetic risk assessment for individuals who are not at increased risk based on family history or clinical presentation is generally restricted to newborn screening programs meeting specific criteria [5,93]. Is there adequate evidence to suggest that a departure from these standards will be beneficial to society? It appears that medical genetics is shifting from the present “phenotype-first” medical model to a “data-first” model which leads to multiple complexities. This manuscript discusses a “phenotype-first” approach, namely, Individualized Mutation-weighed Phenotype Search (I-MPOS), which could potentially render widespread use of ES/WGS, in the immediate future, practical, ethical and clinically useful. The proposed approach allows for a more efficient prioritization of the genes to be tested in a clinical lab based on both the patient’s phenotype and his/her entire genomic data. I-MPOS protects the patient’s privacy and autonomy and enables an incremental integration of genomic technologies into clinical practice. It allows for progressive education of both the health care personnel, including medical geneticists and clinical laboratory scientists, and the general public regarding personalized/preventive medicine. The I-MPOS approach ensures that we do not over-medicalize genomic data but rather continue our current medical model which is based on serving the patient’s concerns. Service should not be solely driven by technology but rather by the medical needs and the extent to which a technology can be safely and effectively utilized.

The era of genomic medicine is already a reality.

## Abbreviations

ES, Exome Sequencing; WGS, Whole Genome Sequencing; I-MPOS, Individualized Mutation-weighed Phenotype On-line Search; I-MPOSE, Individualized Mutation-weighed Phenotype On-line Search Engine.

## Competing interests

Patent application in process: my intention is to ensure that the I-MPOS paradigm can be properly implemented in a timely fashion. As long as the necessary academic collaborations for the programme are available, non-commercial applications of this method will remain free of charge.

## Pre-publication history

The pre-publication history for this paper can be accessed here:

http://www.biomedcentral.com/1755-8794/5/31/prepub
